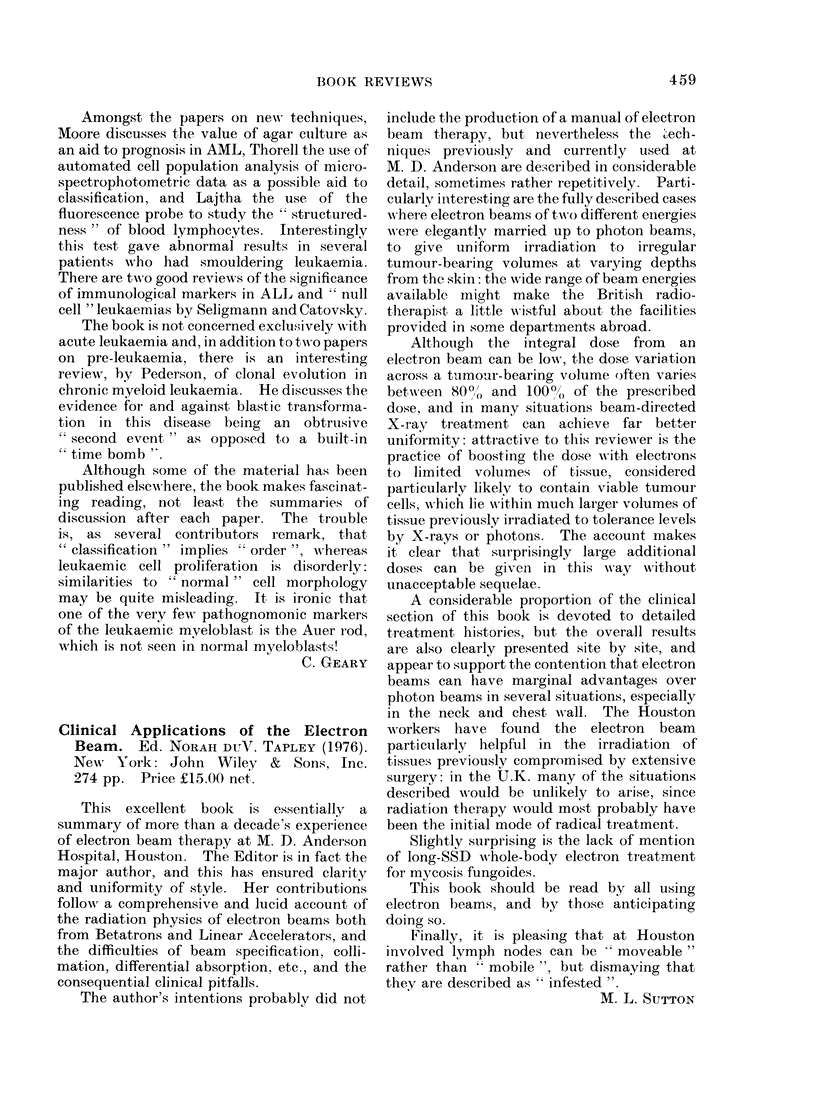# Clinical Applications of the Electron Beam

**Published:** 1976-10

**Authors:** M. L. Sutton


**Clinical Applications of the Electron Beam.** Ed. Norah duV. Tapley (1976). New York: John Wiley & Sons, Inc. 274 pp. Price £15.00 net.

This excellent book is essentially a summary of more than a decade's experience of electron beam therapy at M. D. Anderson Hospital, Houston. The Editor is in fact the major author, and this has ensured clarity and uniformity of style. Her contributions follow a comprehensive and lucid account of the radiation physics of electron beams both from Betatrons and Linear Accelerators, and the difficulties of beam specification, collimation, differential absorption, etc., and the consequential clinical pitfalls.

The author's intentions probably did not include the production of a manual of electron beam therapy, but nevertheless the techniques previously and currently used at M. D. Anderson are described in considerable detail, sometimes rather repetitively. Particularly interesting are the fully described cases where electron beams of two different energies were elegantly married up to photon beams, to give uniform irradiation to irregular tumour-bearing volumes at varying depths from the skin: the wide range of beam energies available might make the British radiotherapist a little wistful about the facilities provided in some departments abroad.

Although the integral dose from an electron beam can be low, the dose variation across a tumour-bearing volume often varies between 80% and 100% of the prescribed dose, and in many situations beam-directed X-ray treatment can achieve far better uniformity: attractive to this reviewer is the practice of boosting the dose with electrons to limited volumes of tissue, considered particularly likely to contain viable tumour cells, which lie within much larger volumes of tissue previously irradiated to tolerance levels by X-rays or photons. The account makes it clear that surprisingly large additional doses can be given in this way without unacceptable sequelae.

A considerable proportion of the clinical section of this book is devoted to detailed treatment histories, but the overall results are also clearly presented site by site, and appear to support the contention that electron beams can have marginal advantages over photon beams in several situations, especially in the neck and chest wall. The Houston workers have found the electron beam particularly helpful in the irradiation of tissues previously compromised by extensive surgery: in the U.K. many of the situations described would be unlikely to arise, since radiation therapy would most probably have been the initial mode of radical treatment.

Slightly surprising is the lack of mention of long-SSD whole-body electron treatment for mycosis fungoides.

This book should be read by all using electron beams, and by those anticipating doing so.

Finally, it is pleasing that at Houston involved lymph nodes can be " moveable " rather than " mobile ", but dismaying that they are described as " infested ".

M. L. Sutton